# Patients with type 1 diabetes mellitus have impaired IL-1β production in response to *Mycobacterium tuberculosis*

**DOI:** 10.1007/s10096-017-3145-y

**Published:** 2017-11-30

**Authors:** E. Lachmandas, K. Thiem, C. van den Heuvel, A. Hijmans, B. E. de Galan, C. J. Tack, M. G. Netea, R. van Crevel, J. A. van Diepen

**Affiliations:** 1 Department of Internal Medicine (463), Radboud Institute for Molecular Life Sciences, Radboud University Medical Centre, Geert Grooteplein zuid 8, 6525 GA Nijmegen, The Netherlands; 2 Radboud Centre for Infectious Diseases, Radboud University Medical Centre, Nijmegen, The Netherlands; 3 Department for Genomics & Immunoregulation, Life and Medical Sciences Institute (LIMES), University of Bonn, 53115 Bonn, Germany

## Abstract

Patients with diabetes mellitus have an increased risk of developing tuberculosis. Although the underlying mechanism is unclear, evidence suggests a role for chronic hyperglycaemia. We examined the influence of hyperglycaemia on *Mycobacterium tuberculosis*-induced cytokine responses in patients with type 1 diabetes mellitus (T1D). Peripheral blood mononuclear cells (PBMCs) from 24 male T1D patients with sub-optimal glucose control [HbA1c > 7.0% (53 mmol/L)] and from 24 age-matched male healthy controls were stimulated with *M. tuberculosis* lysate. Cytokine analysis, assessment of aerobic glycolysis, receptor recognition and serum cross-over experiments were performed to explore the mechanistic differences. PBMCs from T1D patients produced less bioactive interleukin (IL)-1β in response to *M. tuberculosis*. IL-6 and interferon (IFN)-γ production trended towards a decrease, whilst other cytokines such as tumour necrosis factor (TNF)-α, IL-17 and IL-1Ra were normal. The decrease in cytokine production was not correlated to HbA1c or plasma glucose levels. Cross-over serum experiments did not alter the cytokine profile of T1D or control patients, arguing for an intrinsic cellular defect. Cellular metabolism and the expression of *M. tuberculosis*-related pattern recognition receptors (PRRs) such as TLR2, TLR4 and NOD2 did not differ between T1D patients and healthy controls. Compared to matched controls, T1D patients have a reduced capacity to produce pro-inflammatory cytokines in response to *M. tuberculosis*. The impaired IL-1β production in T1D patients may contribute to the increased susceptibility to tuberculosis. This effect appears not to be related to prevailing glucose levels but to an intrinsic cellular deficit.

**Electronic supplementary material:**

The online version of this article (10.1007/s10096-017-3145-y) contains supplementary material, which is available to authorized users.

## Introduction

Diabetes increases the risk of developing active tuberculosis (TB) and is associated with worsened outcomes during TB treatment. It has been estimated that 15% of TB cases globally can be attributed to diabetes [1]. Patients with type 1 diabetes (T1D) may have an even higher risk of developing TB compared to those with type 2 diabetes (T2D) [2–4]. Additionally, poor glucose control escalates the risk of TB [5, 6]. The epidemiological evidence for the relation between diabetes and TB is strong, but the molecular and immunological basis for the susceptibility to TB remains largely unclear.

Recent evidence that points towards a disturbed innate and adaptive immune response to TB is mainly derived from studies in T2D patients [7]. However, T2D is a multi-factorial disease involving age, obesity, sedentary lifestyle and genetics. It is characterised by hyperglycaemia, insulin resistance, hypertension, dyslipidaemia and oxidative stress. All these factors, including the use of anti-diabetic drugs with potential immune-modulating capacities (i.e. metformin), make it difficult to specifically examine the role of hyperglycaemia in the increased susceptibility to TB.

Therefore, we sought to study the response to *Mycobacterium tuberculosis* in T1D patients with chronic hyperglycaemia. We excluded individuals using drugs other than insulin replacement therapy and also those with serious diabetic complications. We examined whether T1D is associated with altered production of pro-inflammatory cytokines such as tumour necrosis factor TNF, interleukin (IL)-1β and IL-6 from monocytes and interferon (IFN)-γ from CD4+ lymphocytes, all of which are pivotal for effective host defences against TB [8]. Finally, we examined whether external factors in serum (i.e. hyperglycaemia) or intrinsic factors (i.e. cellular metabolism and expression of pattern recognition receptors, PRRs) were responsible for differences in cytokine responses between healthy controls and T1D patients.

## Materials and methods

### Recruitment and characterisation of study subjects

We enrolled 24 male T1D patients with an HbA1c > 7.0% (53 mmol/L) and 24 age-matched male healthy controls. Participants were all between 20 and 70 years old. For T1D patients, the minimal duration of diabetes was 1 year. Patients using medication other than insulin were excluded. HbA1c was measured by standard laboratory methods. Plasma insulin was measured by radioimmunoassay [9]. Plasma cholesterol, triglyceride (TG), glucose (Liquicolor; Human GmbH, Wiesbaden, Germany) and free fatty acids (NEFA C; WAKO Chemicals, GmbH, Neuss, Germany) were measured enzymatically following the manufacturers’ protocols. Blood was drawn from a cubital vein and collected into sterile EDTA tubes for isolation of peripheral blood mononuclear cells (PBMCs) or from serum tubes (BD Biosciences, Franklin Lakes, NJ, USA). The study was approved by the institutional review board and written informed consent was obtained from all subjects. Using similar criteria, an additional six T1D and six controls were recruited for a follow-up experiment.

### PBMC isolation and stimulation

PBMC isolation was performed by dilution of blood in pyrogen-free phosphate-buffered saline (PBS) and differential density centrifugation over Ficoll-Paque (GE Healthcare, Zeist, The Netherlands). Cells were washed twice in PBS and re-suspended in RPMI culture medium (Roswell Park Memorial Institute medium; MP Biomedicals, Santa Ana, CA, USA) supplemented with 5 mM glucose, 10 μg/mL gentamicin, 10 mM L-glutamine and 10 mM pyruvate. PBMCs were counted with a Coulter counter (Beckman Coulter, Fullerton, CA, USA) and adjusted to 5 × 10^6^ cells/mL. A 100-μL volume was added to round-bottom 96-well plates (Corning, New York, USA) for PBMC stimulation experiments. Excess unstimulated PBMCs were lysed in TRIzol reagent (Invitrogen, Breda, The Netherlands) and stored at − 80 °C until RNA isolation was performed.

Cells were stimulated with RPMI, 1 μg/mL *M. tuberculosis* (H37Rv) lysate for 24 h or 7 days (in the presence of 10% human pool serum for lymphocyte-derived cytokines). For serum cross-over experiments, cells were incubated with 25% serum for 24 h or 7 days. In a follow-up experiment, we collected six additional male T1D patients with an HbA1c > 7.0% and six age-matched male healthy controls. PBMCs were isolated and stimulated with *Escherichia coli* lipopolysaccharide (LPS) (from *E. coli* serotype 055:B5; Sigma-Aldrich, St. Louis, MO, USA; 1 ng/mL or 10 ng/ml). Supernatants were collected and stored at − 20 °C until cytokine/lactate measurements were performed.

### Cytokine measurements

Cytokine measurements from cell culture supernatants were performed by enzyme-linked immunosorbent assay (ELISA); namely, IL-1β, IL-1 receptor antagonist (IL-1Ra), TNF-α (R&D Systems, Minneapolis, MN, USA) and IL-6 (Sanquin, Amsterdam, The Netherlands) were measured in the 24-h PBMC stimulation experiments. Supernatants of the 7-day stimulations were used to measure IL-22, IL-17 (R&D Systems) or IFN-γ (Sanquin).

### Bioactive IL-1 assay

Active IL-1 was measured indirectly using the mouse thymoma EL4-NOB1 (NOB1) cell line. NOB1 cells were cultured in RPMI culture medium supplemented with 1 mM pyruvate, 1 mM GlutaMAX, 1 mM penicillin/streptomycin and 10% foetal bovine serum (Gibco, Burlington, Ontario, Canada) until confluence was reached. NOB1 cells (10^5^ cells/well) were plated in a flat-bottom 96-well plate (Corning). 70 μL (2× dilution) of supernatant from PBMCs of T1D or healthy controls that were stimulated with *M. tuberculosis* were added to each well. Cytokine measurement for murine IL-2 was performed by ELISA (R&D Systems).

### Lactate measurements

Lactate was measured from cell culture supernatants using a coupled enzymatic assay in which lactate was oxidised and the resulting H_2_O_2_ was coupled to the conversion of Amplex® Red reagent to fluorescent resorufin by horseradish peroxidase (HRP). 30 μL of lactate standard or 200-fold diluted sample was added to a black 96-well flat-bottom plate, followed by 30 μL of reaction mix, which consisted of 0.6 μL of 10 U/mL HRP (Sigma-Aldrich), 0.6 μL of 100 U/mL lactate oxidase (Sigma-Aldrich), 0.3 μL of 10 mM Amplex® Red reagent (Life Technologies, Carlsbad, CA, USA) and 28.5 μL PBS. The assay was incubated for 20 min at room temperature (RT) and the fluorescence of resorufin (excitation/emission maxima = 570/585 nm) was measured on a 96-well plate reader (BioTek, Winooski, VT, USA).

### Transcriptional analysis of isolated PBMCs

RNA was isolated from unstimulated PBMCs using TRIzol reagent (Invitrogen), according to the manufacturer’s protocol. RNA was transcribed into complementary DNA by reverse-transcription using the iScript cDNA Synthesis Kit (Bio-Rad, Hercules, CA, USA). Quantitative real-time polymerase chain reaction (qPCR) was performed using different primer sets (Biolegio, Malden, The Netherlands); primer sequences for hexokinase (HK) 2, HK3, 6-phosphofructo-2-kinase/fructose-2,6-biphosphatase 3 (PFKFB3), pyruvate dehydrogenase kinase 4 (PDK4), malate dehydrogenase (MDH) 1, MDH2, toll-like receptor (TLR) 2, TLR4, nucleotide-binding oligomerisation domain containing 2 (NOD2), caspase recruitment domain family member 9 (CARD9), receptor interacting serine/threonine kinase 2 (RIPK2), mitogen-activated protein kinase 9 (MAPK9), TNF receptor-associated factor 6 (TRAF6) and caspase 1 (CASP-1) are given in Supplementary Table 1. Power SYBR Green PCR Master Mix (Applied Biosystems, Carlsbad, CA, USA) was used for qPCR in a StepOnePlus Real-Time PCR System (Applied Biosystems). qPCR data were normalised to the housekeeping gene human β2M.

### Statistics

Data are shown as means ± standard error of the mean (SEM). Differences in cytokine secretion were calculated using the Mann–Whitney test for two independent samples. Correlation analysis between glucose metabolites and cytokine secretion was performed using Spearman’s rank correlation coefficient and the 95% confidence interval was calculated accordingly. A *p*-value of < 0.05 was considered statistically significant. Generation of graphs and statistical analyses were performed using GraphPad Prism 5.

## Results

The participant’s characteristics are shown in Table 1. Age, plasma TG and free fatty acid (FFA) levels were not different between T1D patients and the 24 control subjects (Table 1). As per definition, HbA1c and plasma glucose levels were significantly increased in T1D patients compared to healthy controls. Plasma insulin levels were also higher, whilst plasma cholesterol levels were lower in T1D patients.

**Table 1:** Descriptive characteristics of the studied type 1 diabetes (T1D) patients and controls

	T1D patients	Controls	*p*-Value
Number	24	24	
Age (years)	48.1 ± 2.7	47.5 ± 2.6	0.91
Duration of diabetes (years)	24.2 ± 2.5	–	–
Glucose (mmol/L)	9.3 ± 0.8	4.6 ± 0.2	< 0.001***
HbA1c (%)	8.9 ± 0.3	5.4 ± 0.1	< 0.0001***
Insulin (mE/L)	31.5 ± 3.8	19.9 ± 2.9	< 0.05*
Cholesterol (mmol/L)	3.5 ± 0.1	4.0 ± 0.2	0.05
TG (mmol/L)	1.36 ± 0.15	1.25 ± 0.12	0.70
FFA (mmol/L)	0.25 ± 0.04	0.25 ± 0.04	0.39

Data are mean ± standard error of the mean (SEM). FFA, free fatty acids; HbA1c, glycosylated haemoglobin; TG, triglyceride

### PBMCs of T1D patients show reduced pro-inflammatory IL-1β cytokine secretion in response to *M. tuberculosis*

**Fig. 1:**
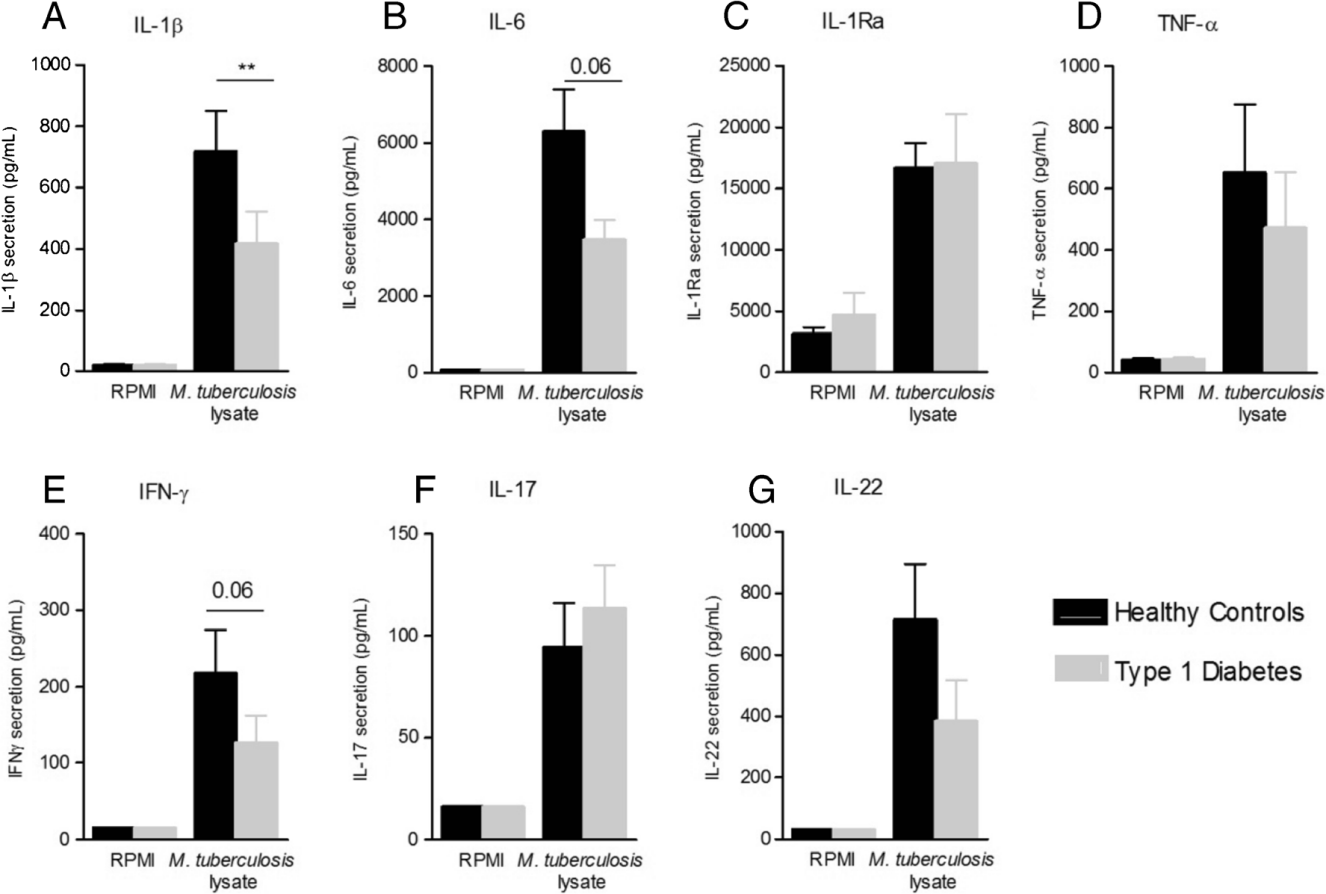
Peripheral blood mononuclear cells (PBMCs) from type 1 diabetes mellitus (T1D) subjects secrete less interleukin (IL)-1β upon stimulation with *Mycobacterium tuberculosis*. PBMCs (5 × 10^5^/well) from T1D patients and healthy control subjects were stimulated with 1 μg/mL *M. tuberculosis* lysate. Secretion of: **a** IL-1β, **b** IL-6, **c** IL-1Ra and **d** tumour necrosis factor (TNF)-α were measured in supernatants by enzyme-linked immunosorbent assay (ELISA) after 24 h of stimulation. **e** Interferon (IFN)-γ, **f** IL-17 and **g** IL-22 were measured after 7 days of stimulation. Data are mean ± standard error of the mean (SEM) from *n* = 24 individuals per group. **p < 0.01 compared with matched healthy controls

No spontaneous cytokine production was detected in unstimulated TID patients or control cells (RPMI; Fig. 1). In contrast, robust induction of cytokine production was observed in response to *M. tuberculosis* in both groups. The production of IL-1β was significantly lower in T1D patients as compared to healthy controls (*p* < 0.01; Fig. 1). IL-6 and IFN-γ followed a similar trend, albeit at borderline significance (*p* = 0.06). TNF-α, IL-1Ra, IL-17 and IL-22 levels were not different between T1D patients and controls (Fig. 1). The decrease in IL-1β production was not specific to *M. tuberculosis* stimulation. Six additionally recruited T1D patients also produced lower levels of IL-1β in response to high-dose LPS stimulation (Supplementary Fig. 1).

### Correlation of IL-1β cytokine secretion with glycaemia

**Fig. 2:**
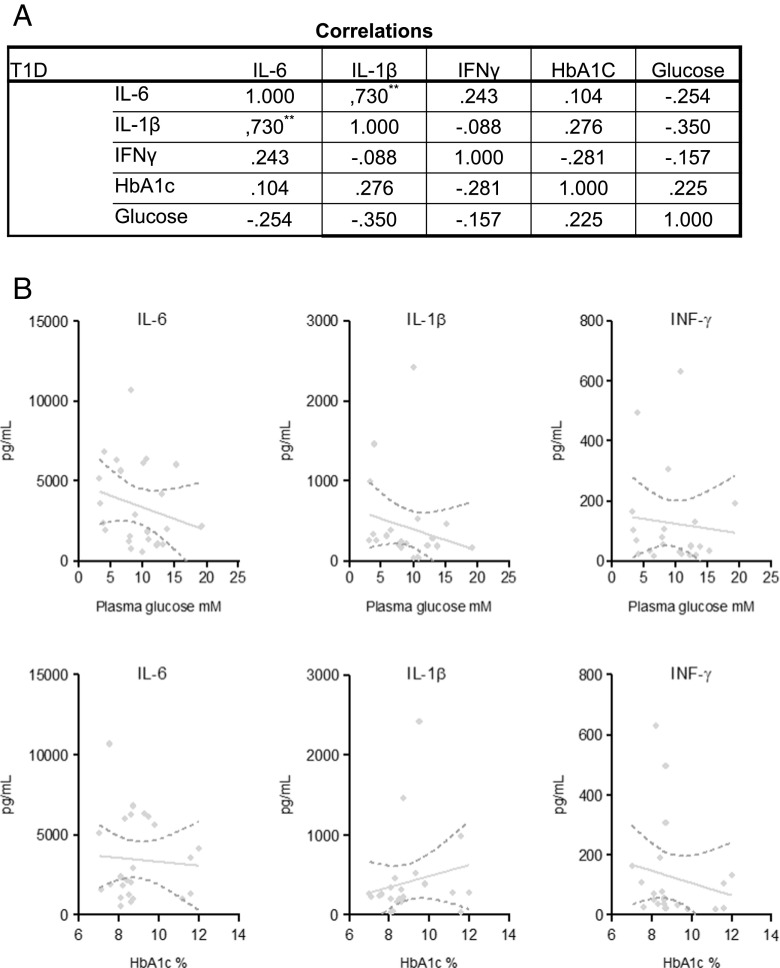
Correlation of cytokine secretion with glycaemia. Associations for T1D patients between glycated haemoglobin (HbA1c%), plasma glucose levels and secretion of IL-1β, IL-6 and IFN-γ from PBMCs after stimulation with 1 μg/mL *M. tuberculosis* lysate. **a** Spearman’s rank correlation coefficients for all associations. **b** Correlations are shown by linear fitted curves and 95% confidence intervals for T1D subjects (*n* = 24)

To determine whether the lower cytokine production in PBMCs from T1D patients in response to *M. tuberculosis* stimulation was related to glucose control, cytokine production was correlated with HbA1c and glucose. However, there were no correlations between *M. tuberculosis*-induced IL-1β, IL-6 or IFN-γ secretion from PBMCs and HbA1c or plasma glucose levels of T1D patients (Fig. 2). Also, no correlations were found between cytokine secretion and duration of diabetes (Supplementary Fig. 2). IL-1β secretion strongly correlated with IL-6 secretion in response to *M. tuberculosis*, but there were no other correlations between cytokine responses.

### Influence of serum and cellular metabolism on cytokine production

**Fig. 3:**
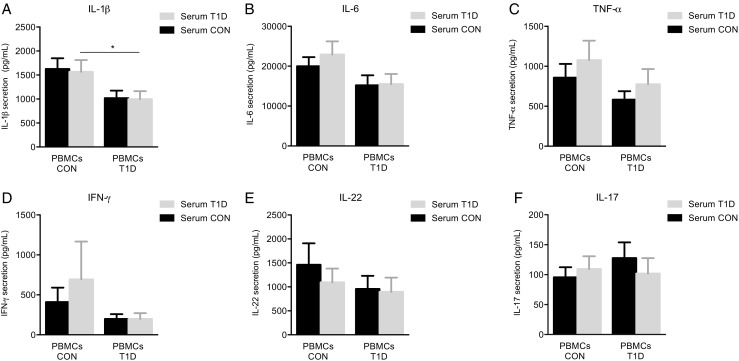
Influence of T1D serum on cytokine production in response to *M. tuberculosis*. PBMCs (5 × 10^5^ cells/well) of healthy control (*CON*) subjects were incubated in either 25% of autologous serum or 25% of serum from an age-matched type 1 diabetes (*T1D*) subject and vice versa for PBMCs from T1D subjects. Secretion of: **a** IL-1β, **b** IL-6 and **c** TNF-α were measured in the supernatants using ELISA after 24 h of exposure to *M. tuberculosis*. **d** INF-γ, **e** IL-22 and **f** IL-17 were determined after 7 days. Data are mean ± SEM from *n* = 24 individuals per group. *p < 0.05 compared with healthy controls

The cross-over of autologous serum from control to T1D and vice versa did not influence cytokine production of either subject group (Fig. 3). This suggests that the reduced IL-1β secretion is not directly related to plasma glucose concentration or other serum factors; instead, it is likely to be an intrinsic defect within the immune cells of T1D patients.

**Fig. 4:**
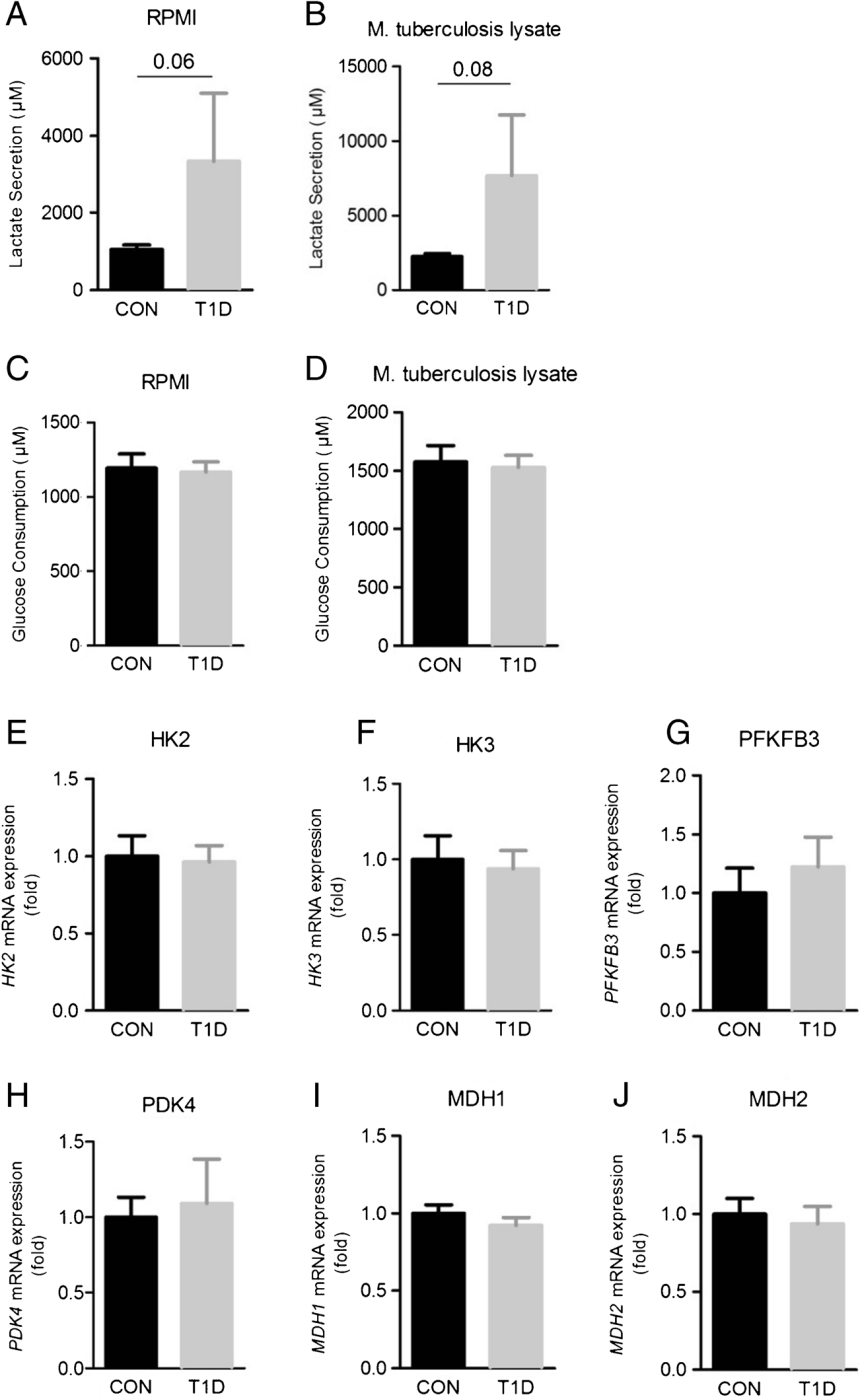
Cellular metabolism in PBMCs from T1D subjects and controls. **a** Lactate secretion and **c** glucose consumption were measured in the supernatant of unstimulated (RPMI) PBMCs of type 1 diabetes (*T1D*) and healthy control (*CON*) subjects as well as **b**, **d** after 24 h of stimulation with *M. tuberculosis* lysate. Relative mRNA expression values of **e** HK2, **f** HK3, **g** PFKFB3, **h** PDK4, **i** MDH1 and **j** MDH2 in unstimulated PBMCs from T1D and CON subjects. Data are mean ± SEM from *n* = 24 individuals per group

Activation of aerobic glycolysis is important for cytokine production in response to *M. tuberculosis* [10]. However, lactate production, a marker of glycolysis, was not decreased in T1D patients. In fact, *M. tuberculosis* stimulation increased lactate production in T1D patients compared to matched healthy controls (Fig. 4a). Glucose consumption (Fig. 4b) and the expression levels of glycolysis genes HK2, HK3 and PFKFB3 in PBMCs was similar between T1D patients and healthy controls (Fig. 4e–g). Finally, no difference in the expression of the TCA cycle genes PDK4, MDH1 and MDH2 (Fig. 4h–j) was observed.

### *Mycobacterium tuberculosis* recognition and downstream signalling

**Fig. 5:**
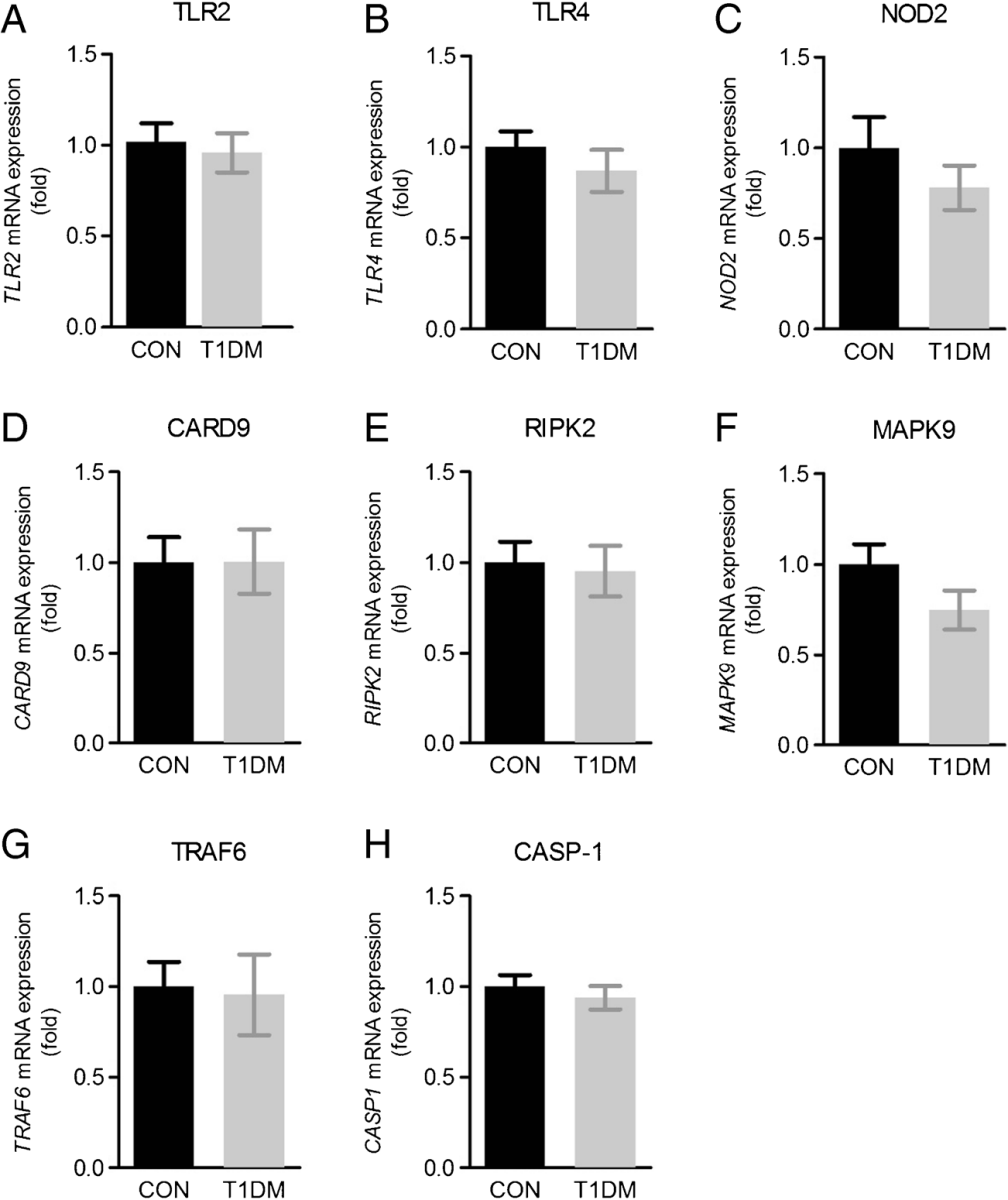
*Mycobacterium tuberculosis* recognition and downstream signalling. Relative basal mRNA expression values of PBMCs from type 1 diabetes (*T1D*) patients are shown when compared to matched healthy controls (*CON*) for **a** TLR2, **b** TLR4, **c** NOD2, **d** CARD9, **e** RIPK2, **f** MAPK9, **g** TRAF6 and **h** CASP1. Data are mean ± SEM from *n* = 24 individuals per group

To determine whether changes in receptors involved in *M. tuberculosis* recognition could explain the impaired cytokine response of PBMCs from T1D patients, we investigated the gene expression levels of well-known *M. tuberculosis* PRRs in PBMCs of both groups. Levels of TLR2, TLR4 and NOD2 were unchanged (Fig. 5a–c). In addition, no differences were found in the expression of genes involved in the intracellular signalling response to *M. tuberculosis*, including CARD9, RIPK2, MAPK9, TRAF6 and CASP1 (Fig. 5d–h).

### Bioactive IL-1 secretion from PBMCs of T1D patients

**Fig. 6:**
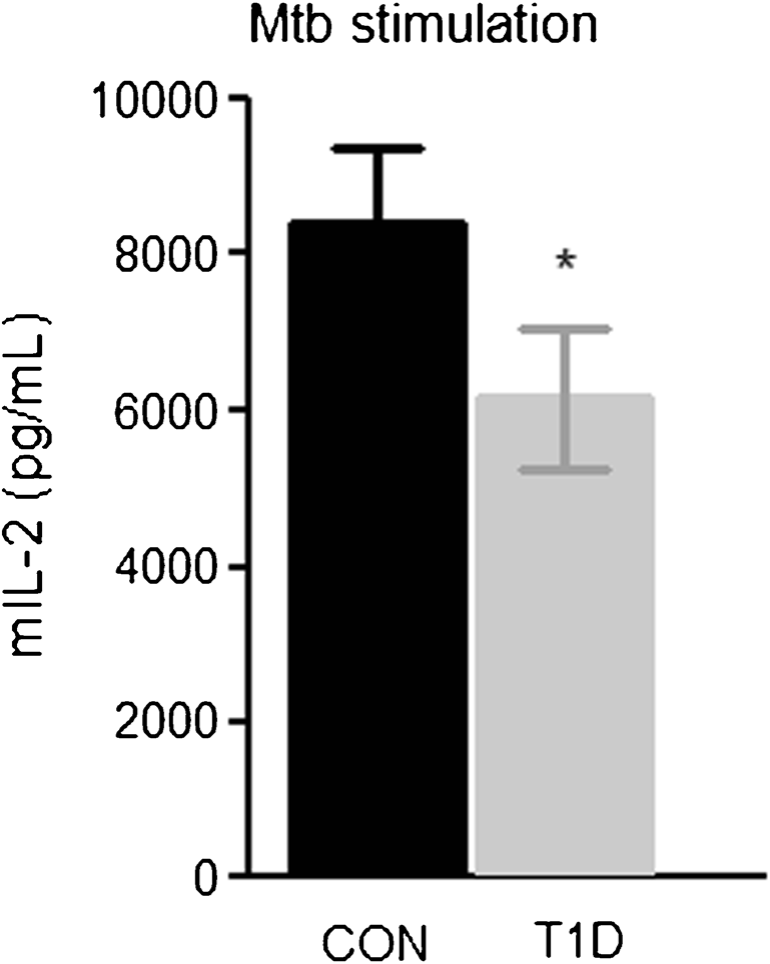
Bioactive IL-1 secretion from PBMCs of T1D patients. PBMCs (5 × 10^5^/well) from type 1 diabetes patients (*T1D*) and healthy control subjects (*CON*) were stimulated with 1 μg/mL *M. tuberculosis* lysate for 24 h and supernatants were subsequently incubated with the murine thymoma EL4-NOB1 cell line (10^5^ cells/well). Mouse IL-2 cytokine secretion in response to present bioactive IL-1 in the supernatant of the PBMCs was measured using ELISA. Data are mean ± SEM from *n* = 24 individuals per group. *p < 0.05 compared with the matched healthy controls

To determine whether differences in cellular processing affected the secretion of IL-1β, we determined the amount of bioactive IL-1 in the supernatants of PBMCs stimulated with *M. tuberculosis* from Fig. 1 (Fig. 6). We found that PBMCs of T1D patients released significantly lower levels of bioactive IL-1 in response to *M. tuberculosis* .

## Discussion

The primary finding of this study is that PBMCs from patients with T1D have a reduced ability to produce IL-1β, IL-6 and IFN-γ in response to *M. tuberculosis* stimulation, while TNF, IL-17 and IL-1Ra production is normal. These changes may be partly responsible for the well-known susceptibility of patients with diabetes for TB. The current data suggest that the decreased cytokine production after stimulation with *M. tuberculosis* is an intrinsic cellular deficit in PBMCs from T1D patients, since differences in cytokine secretion were not due to external factors in serum or related to glucose regulation.

Epidemiological studies that investigated the relation between hyperglycaemia and the risk for TB have been contradicting. Both a positive effect [5, 6] and no effect [11–13] of hyperglycaemia on the risk for TB have been described. These studies vary greatly in size, geography and definition of controlled versus uncontrolled glycaemia. To our knowledge, this is the first study specifically examining the cytokine production capacity of T1D patients to *M. tuberculosis*. At the same time, studies on the cytokine response to other pathogenic stimuli such as LPS strongly support our findings. Ex vivo stimulation experiments performed in both T1D [14–16] and T2D [17, 18] patients describe a similar decrease of IL-1β and IL-6 but not TNF-α, IL-1Ra and IFN-γ production, respectively. As shown in the stimulation experiments with LPS, this decrease in cytokine signalling is not specific for *M. tuberculosis*, thus supporting the notion that an intrinsic defect in cytokine production rather than an *M. tuberculosis*-specific signalling defect is the mechanism underlying the decrease in cytokine production. This may also explain why T1D patients are also susceptible to other (common) infections, such as lower respiratory tract infection, urinary tract infection, and skin and mucous membrane infection [19, 20].

Interestingly, patients with both TB and diabetes produce more cytokines compared to patients with only TB [21, 22]. Together, these findings suggest an initial sub-optimal response to infection which promotes bacterial growth and subsequent pathology. This hypothesis of a delayed immune response is supported by a mouse model of concomitant TB and diabetes disease [23].

The exact type of intracellular defects remains to be elucidated. No change in aerobic glycolysis was observed nor altered expression of relevant PRRs. It is possible that a certain level of accelerated immunosenescence occurs in T1D. This process, described for aging, is characterised by impaired cellular immune function (leading to increased death due to infectious diseases), simultaneously with increased chronic inflammatory activity (leading to increased incidence of cardiovascular disease) [24]. The observations that T1D patients show enhanced low-grade chronic inflammation and cardiovascular diseases [25] and, at the same time, an impaired immune response to pathogens as observed in the current study support this hypothesis. The decrease in IL-6 may be a secondary effect as IL-1β induces IL-6 production in a paracrine signalling loop. Similarly, IL-18, another product of caspase-1 activity, is important for IFN-γ production. This could explain why the decrease in these cytokines is more subtle as compared to IL-1β. This avenue will form a cornerstone for further research.

The implications of a decrease in IL-1β production for susceptibility to TB are significant. In a murine model of TB, IL-1α/β double knockout mice [26] or mice deficient in IL-1R1 [27] display increased susceptibility to *M. tuberculosis* infection. Although the spectra of human and mouse TB do not completely overlap, there are firm established points of convergence [28]. Therefore, these mouse studies suggest that IL-1 has an important role in the generation of an adequate host defence against TB. In humans, a positive association between IL-1β levels and resistance to TB was inferred from human *IL1B* gene polymorphisms [29]. Moreover, patients with gain-of-function mutations in the inflammasome component NLRP3 have an increased capacity to kill *M. tuberculosis* [30]. Mechanistically, a recent study elucidated that IL-1β promoted bacterial containment through the induction of specific eicosanoids that limited excessive type 1 IFN-γ production [31]. The current study was performed in T1D patients, with chronic hyperglycaemia as a key characteristic. The observed impaired cellular cytokine response could be similar for T2D patients. However, T2D is a much more heterogeneous disease characterised by multiple metabolic disturbances (i.e. hyperglycaemia, insulin resistance, hypertension, dyslipidaemia and oxidative stress), which may further contribute to the increased susceptibility to TB and which needs further investigation.

As a limitation of this study, we did not investigate other host defence mechanisms such as phagocytosis, reactive oxygen species (ROS) or nitric oxide (NO) production from monocytes or macrophages, since it is unlikely that reduced IL-1β is the sole factor in susceptibility to TB. A confounding factor in the current study could be the significantly higher levels of insulin in blood of T1D patients, as insulin can affect cytokine production; however, we did not find any correlation between insulin and cytokine production in T1D patients (Supplementary Fig. 3). Lastly, another limitation of this study is the relatively small size of our cohort, as a result of which subtle associations such as those between glucose control and cytokine responses may not be detectable. We chose to study only male patients and healthy controls in order to reduce variability in the cytokine response as much as possible, hence enabling the generation of robust results with 24 individuals per group. Indeed, for example, oral contraceptive usage in women has been shown to significantly impact on cytokine secretion from PBMCs [32]. Although we cannot completely exclude differences of cytokine production between male and female diabetes patients, no differences have been observed previously between healthy men and women regarding the IL-1β response to *M. tuberculosis* [32], suggesting that our results derived from male participants are most likely relevant for both men and women.

In conclusion, this study specifically identifies a decrease in bioactive IL-1β to possibly be, due to a deficit in intracellular processing rather than chronic hyperglycaemia, a strong candidate in the susceptibility of T1D patients to TB.

## Electronic supplementary material

Below is the link to the electronic supplementary material.

ESM 1 (DOC 1314 kb)
