# Supplementary material for: Patients with type 1 diabetes mellitus have impaired IL-1β production in response to Mycobacterium tuberculosis
Source: Eur J Clin Microbiol Infect Dis. 2017 Nov 30;37(2):371–80. doi: 10.1007/s10096-017-3145-y (PMC5780542; doi:10.1007/s10096-017-3145-y)
Supplement: Supplementary file 1 — (DOC 1314 kb) [file 10096_2017_3145_MOESM1_ESM.doc]

**ELECTRONIC SUPPLEMENTARY MATERIAL**

**Patients with type 1 diabetes mellitus have impaired IL-1β production in response to *Mycobacterium tuberculosis***

European Journal of Clinical Microbiology & Infectious Diseases

Ekta Lachmandas^1,2^, Kathrin Thiem^1^, Corina van den Heuvel^1^, Anneke Hijmans^1^, Bastiaan E. de Galan^1^, Cees J. Tack^1^, Mihai G Netea^1,2,3^, Reinout van Crevel^1,2^ and Janna A. van Diepen^1^

^1^Department of Internal Medicine, Radboud Institute for Molecular Life Sciences, ^2^Radboud Centre for Infectious Diseases, Radboud University Medical Centre, Nijmegen, The Netherlands, ^3^Department for Genomics & Immunoregulation, Life and Medical Sciences Institute (LIMES), University of Bonn, 53115 Bonn, Germany

**^#^ Corresponding author:**

Janna A. van Diepen
Department of Internal Medicine (463), Radboud University Medical Centre, Geert Grooteplein zuid 8, 6525 GA, Nijmegen, The Netherlands
Phone: +31-243610244
E-mail: [Janna.vanDiepen@radboudumc.nl](mailto:Janna.vanDiepen@radboudumc.nl)

**Supplementary Table 1** Primer sequences used for qRT-PCR analysis (5’-3’)


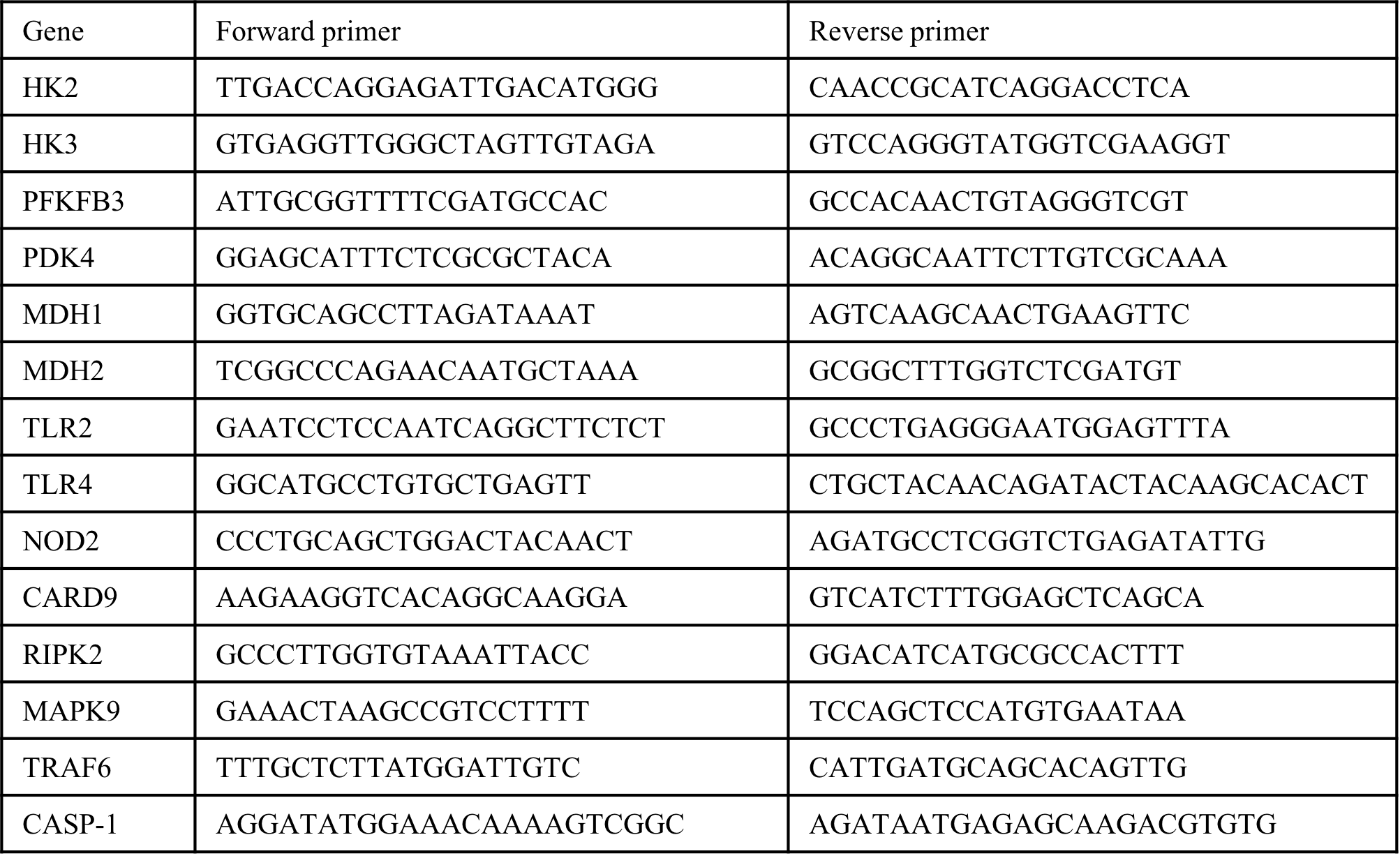


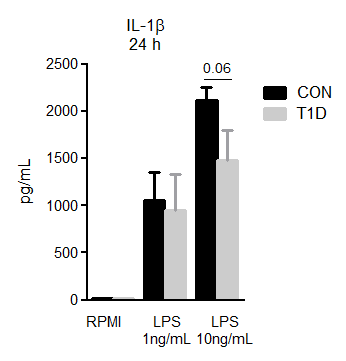


**Supplementary Fig1 Reduced IL-1b secretion by PBMCs from T1D subjects is not specific to M. *tuberculosis* stimulation** Blood peripheral mononuclear cells (PBMCs) of six additional type 1 diabetes (T1D) subjects and healthy control (CON) subjects have been challenged with low and high dose of lipopolysaccharide (LPS, 1ng/mL and 10ng/mL) for 24 h and interleukin (IL)-1b secretion was measured using ELISA. Data are mean ± SEM from n=6 individuals per group.


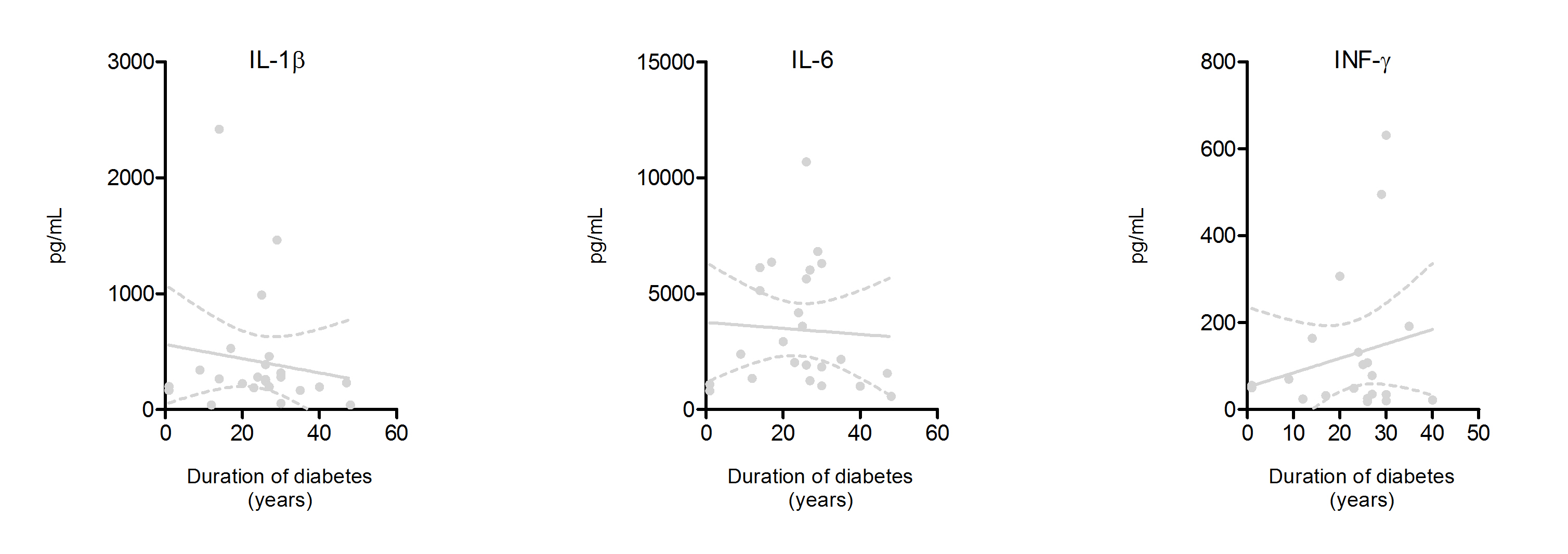


**Supplementary Fig2 Correlation of cytokine secretion with duration of diabetes** Associations for T1D patients between duration of diabetes (years) and secretion of interleukin (IL)-1β, IL-6 and interferon (IFN)-y from PBMCs after stimulation with 1 µg/mL M. *tuberculosi*s lysate. Correlations are shown by linear fitted curves and 95% confident intervals for T1D subjects (n=24).


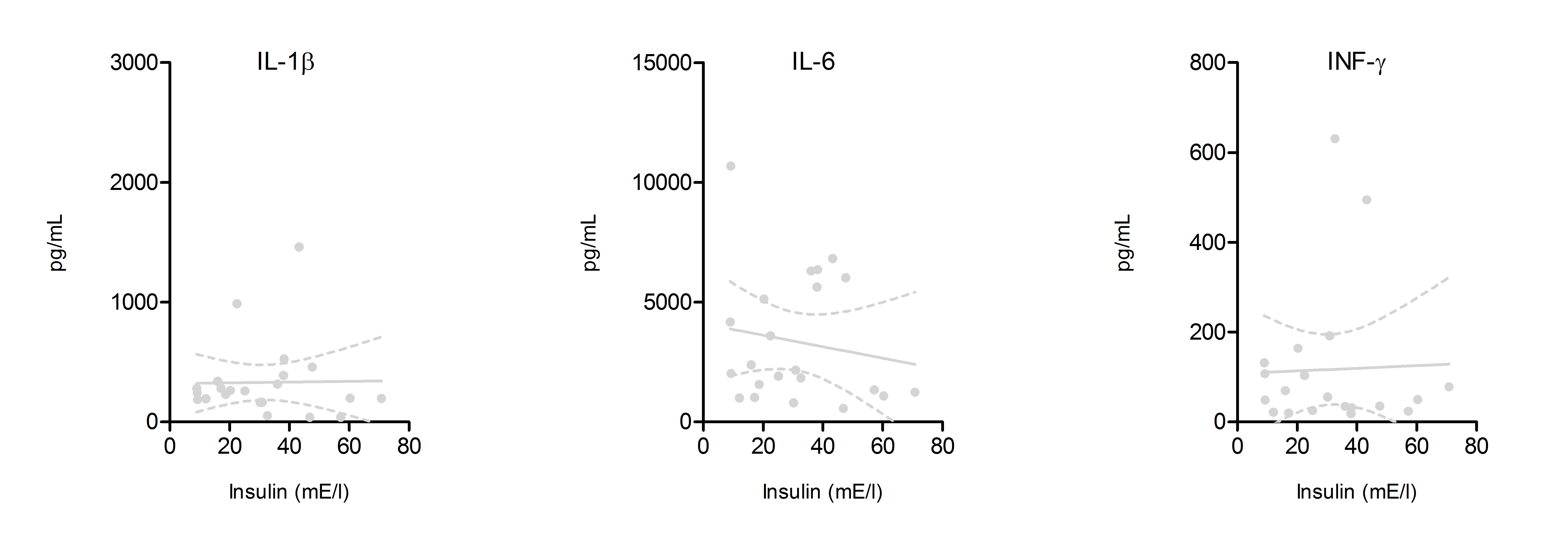


**Supplementary Fig3 Correlation of cytokine secretion with plasma insulin levels** Associations for T1D patients between plasma insulin levels (mE/l) and secretion of interleukin (IL)-1β, IL-6 and interferon (IFN)-y from PBMCs after stimulation with 1 µg/mL M. *tuberculosi*s lysate. Correlations are shown by linear fitted curves and 95% confident intervals for T1D subjects (n=24).
